# Endothelial to mesenchymal transition: at the axis of cardiovascular health and disease

**DOI:** 10.1093/cvr/cvae021

**Published:** 2024-02-22

**Authors:** Ignacio Fernando Hall, Franceska Kishta, Yang Xu, Andrew H Baker, Jason C Kovacic

**Affiliations:** Centre for Cardiovascular Science, Queens Medical Research Institute, University of Edinburgh, Edinburgh EH16 4TJ, UK; Centre for Cardiovascular Science, Queens Medical Research Institute, University of Edinburgh, Edinburgh EH16 4TJ, UK; Cardiovascular Research Institute, Icahn School of Medicine at Mount Sinai, 1 Gustave L. Levy Place, New York, NY 10029, USA; Centre for Cardiovascular Science, Queens Medical Research Institute, University of Edinburgh, Edinburgh EH16 4TJ, UK; CARIM School for Cardiovascular Diseases, Maastricht University, Maastricht 6229ER, The Netherlands; Cardiovascular Research Institute, Icahn School of Medicine at Mount Sinai, 1 Gustave L. Levy Place, New York, NY 10029, USA; Victor Chang Cardiac Research Institute, Lowy Packer Building, 405 Liverpool Street, Darlinghurst, NSW 2010, Australia; St. Vincent’s Clinical School and University of New South Wales, 390 Victoria St, Darlinghurst, NSW 2010, Australia

**Keywords:** Endothelial-to-mesenchymal transition (EndMT), Development, Human disease, Endothelial cell biology, Cellular plasticity and heterogeneity, Therapeutic options

## Abstract

Endothelial cells (ECs) line the luminal surface of blood vessels and play a major role in vascular (patho)-physiology by acting as a barrier, sensing circulating factors and intrinsic/extrinsic signals. ECs have the capacity to undergo endothelial-to-mesenchymal transition (EndMT), a complex differentiation process with key roles both during embryonic development and in adulthood. EndMT can contribute to EC activation and dysfunctional alterations associated with maladaptive tissue responses in human disease. During EndMT, ECs progressively undergo changes leading to expression of mesenchymal markers while repressing EC lineage-specific traits. This phenotypic and functional switch is considered to largely exist in a continuum, being characterized by a gradation of transitioning stages. In this report, we discuss process plasticity and potential reversibility and the hypothesis that different EndMT-derived cell populations may play a different role in disease progression or resolution. In addition, we review advancements in the EndMT field, current technical challenges, as well as therapeutic options and opportunities in the context of cardiovascular biology.

## 1. Introduction

**Figure 1:**
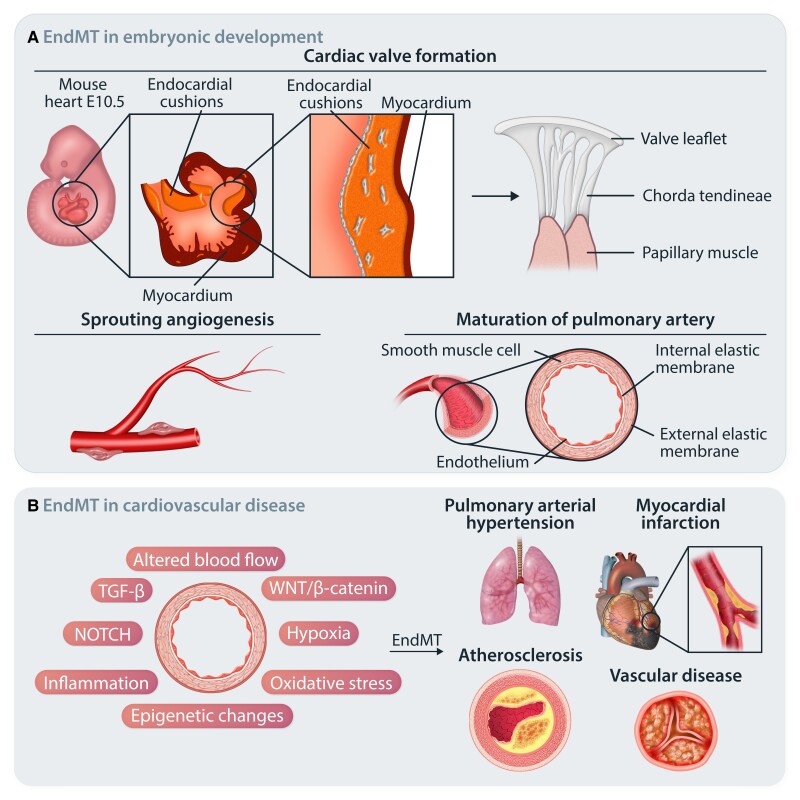
EndMT in health and disease. (*A*) Schematic representation of EndMT in embryonic development. A subset of ECs lining the AV canal undergo EndMT and mediate endocardial cushion formation. Further endocardial cushion remodelling mediates healthy AV valve formation. Developmental EndMT is also reported in sprouting angiogenesis and in the maturation of pulmonary arteries. (*B*) In adult life, EndMT has been associated with the onset and progression of numerous cardiovascular diseases and certain malignancies.

The vascular endothelium is a major regulator of vessel biology in health and disease. While the importance of the endothelium has long been recognized to be at the axis of health and disease,^1^ important breakthroughs have been made over the last decades.^2–5^ Recent studies demonstrate that endothelial cells (ECs) show great plasticity and heterogeneity, as underscored by their highly specialized functions across different organs and vascular beds.^6,7^ Activated ECs have the capacity to undergo endothelial-to-mesenchymal transition (EndMT), a complex biological process defined by the progressive and dynamic loss of endothelial phenotype concurrent with the acquisition of a mesenchymal profile.^8–11^ EndMT is fundamental for atrioventricular (AV) valve formation in developmental cardiogenesis and is widely recognized to promote cellular trans-differentiation or transient cellular adaptions.^12–14^ A wide body of research has demonstrated that EndMT is associated with divergent pathologies including malignancy, vascular, inflammatory, and fibrotic diseases,^15–21^ as summarized in *Figure 1*. The precise molecular mechanisms governing EndMT remain, in the main, elusive, particularly in the context of EC fate and transitioning spectrum, the identity of downstream EndMT-derived cell subtypes, process remodelling, and reversal. In this review, the endothelial-to-mesenchymal phenotypic switch will be described both in contexts of cardiovascular development and (patho)-physiological remodelling. We then discuss key signalling pathways, epigenetic mechanisms, and EndMT-associated transcriptional factors (TFs) implicated in such contexts. Additionally, current limitations in EndMT experimental models and in high-throughput techniques are discussed while proposing advances that can reinforce future studies. Despite prevailing limitations, the advancement in the study of EndMT and its biology hold the promise of being new therapeutic avenues in human disease.

## 2. ECs in health and disease

### 2.1 Development vs. adulthood

The vasculature forms early during embryo development and is essential for the growth, survival, and function of all organ systems. Its innermost layer, the endothelium, is a major regulator of vessel biology in health and disease.^5,22^ It consists of a single layer of ECs lining the luminal surface of blood vessels, whereby ECs mediate a complex regulation of the vessel wall. First, embryonic ECs originate from the mesoderm and undergo profound remodelling changes promoting vessel wall formation and organization into a circulatory network.^23^ Subsequently, ECs acquire site-specific specializations to address distinct structural and biological needs in an organ-specific manner and based on their position along the vasculature or within a specific vascular bed.^23–25^

The situation is different in adulthood where healthy ECs are mostly in a quiescent state defined by reduced endothelial proliferation, reduced migration, and maintained barrier integrity.^26,27^ EC homeostasis results from a dynamic balance wherein the endothelium uses paracrine and endocrine factors, alongside autocrine feedback loops, to maintain cell stability and normalcy.^28–30^ The crosstalk between ECs and bystander cells [e.g. vascular smooth muscle cells (VSMCs) and pericytes] is critical, and the disruption of this cell–cell communication (by direct or indirect interaction) negatively affects vessel homeostasis.^31,32^

Far from representing merely a physical barrier, ECs participate in sensing circulating factors and responding to a range of intrinsic and extrinsic signals.^33^ ECs are thereby involved in a wide variety of processes, spanning from regulation of vascular tone, cellular adhesion, migration, coagulation, and vessel wall permeability to inflammatory processes.^11^ However, imbalances in EC homeostatic functions and failure to recover from noxious stimuli can lead to disease, promoting the reactivation of EndMT.^19,20,34,35^ Such activation leads to vascular integrity disruption by affecting EC cell–cell adhesion, promoting metalloproteinase (MMPs)-mediated extracellular matrix (ECM) degradation,^36,37^ and acquisition of migratory and proliferative cellular properties.^38,39^ Inflammatory stress plays a central role both by promoting endothelial dysfunction and predisposing the vasculature towards a pro-inflammatory state, thus establishing a vicious circuit.^40,41^ Indeed, chronic inflammation and sustained EC activation contribute to diseases such as atherosclerosis and pulmonary arterial hypertension (PAH), with accumulating evidence suggesting EndMT is a possible key link between inflammation and EC dysfunction.^20,42,43^

### 2.2 EC heterogeneity: one definition for many cues

Several studies have addressed the remarkable heterogeneity of ECs, highlighting differences in terms of cell structure and organization, which are likely dictated by different embryonic origins and which contribute to EC specialization. This reflects the wide array of functions that ECs perform to maintain cell homeostasis while adapting to site-specific demands.^6,24,44^ The intrinsic plasticity and adaptation capacity of ECs are paramount in determining cell fate changes in pathologic conditions affecting endothelium normalcy.^11,23^ In disease, EC heterogeneity appears to be fairly broad, being defined by factors such as changes in the proportions of cellular subpopulations present both in health and disease, their restricted expression only in an experimental group/condition, as well as changes in the EC interactome and morphological/functional properties.^7^ At the far end of the heterogeneity spectrum, ECs can undergo either transient or permanent cellular fate and functional adaptions, as observed in EndMT where a subset of ECs undergo profound changes.^6,7,45^ It is therefore reasonable to hypothesize that EndMT-responsive EC subsets may be primed to respond to homeostatic disruptions, either by hindering or advancing endothelial dysfunction. Which are the molecular switches guiding this decision and whether they are conserved across EndMT-associated pathologies or present in context-specific scenarios remain to be elucidated, representing the subject of intense investigation.

## 3. EndMT role in EC biology

### 3.1 EndMT process overview

EndMT is a complex biological process characterized by profound morphological, molecular, and functional changes in EC phenotype.^46–48^ EndMT plays an integral role in embryonic development^14,49,50^ and was first described as a locally confined process involving subsets of ECs during cardiogenesis and vasculogenesis.^12,13,49^ These seminal studies showed that cardiac cushion cells, which are the precursors to cardiac valves, originate from a distinct subset of endocardial ECs that transitioned to mesenchymal cells via EndMT.^50,51^ EndMT has also been shown to be involved in the maturation of pulmonary arteries.^16^

A growing body of evidence indicates that EndMT also plays a central role in tissue dysfunction in human adult pathologies, including cancer^15,17,52–54^ and a variety of cardiovascular diseases.^55^ However, it has been reported that EndMT might be beneficial to some extent, being involved also in neovascularization, angiogenesis, tissue repair, and regeneration.^4,56–59^ Overall, post-natal reactivation of EndMT can be considered as a potential mechanism for adaptation to new (patho)-physiological settings, where different roles are possible in virtue of the highly dynamic nature of EndMT.

Despite intense research, many aspects of EndMT biology remain poorly understood, as most of the mechanistic insight on this phenomenon originates from the study of epithelial-to-mesenchymal transition (EMT).^11,50,60^ EMT is a well-studied process, known to be evolutionary conserved and induced by a broad spectrum of stressors, including cytokines, mechanical forces, and metabolic factors. While EMT is required for normal embryonic development, it can be hijacked in pathological conditions to facilitate tissue fibrosis and cancer metastasis.^46,60^ Although our understanding of EndMT is more limited than for EMT, the endothelium is a specialized sub-type of epithelium, thus affording the possibility to extend some of the prior knowledge regarding EMT to EndMT.^11^ In EndMT biology, one of the main limitations is the lack of a consensus of an exact molecular and functional definition. As the expression of EndMT-associated markers varies with time and environment, EndMT assessment and data cross-comparability can be extremely challenging.^20,43,61^ Despite these challenges, EndMT is determined by an increased expression of mesenchymal markers and the concurrent decrease in endothelial ones at transcript/protein level.^8,10^ EndMT hallmarks consist of the myogenic proteins α-smooth muscle actin (α-SMA) and transgelin (TAGLN/SM22α), non-myogenic fibroblast markers such as fibroblast-specific protein-1 (FSP-1), and fibrillary collagens type I and type III (COL1A1 and COL3A1), with many others being reported in context- or stimulus-specific responses. On the other hand, EC lineage-specific markers negatively modulated during EndMT include platelet–endothelial cell adhesion molecule-1 (PECAM1/CD31), von Willebrand factor (VWF), and vascular–endothelial cadherin (VE-cadherin/CDH5). As already appreciated from the study of EMT, the EndMT programme is also orchestrated by TFs such as twist-related protein-1 (TWIST), the Mothers Against Decapentaplegic Homolog (SMAD) family member 3 (SMAD3), the zinc finger E-box-binding homeobox 2 (ZEB2), and the Snail family transcriptional repressors 1 and 2 (SNAI1, SNAI2).^52,62–66^ It is worth mentioning that studies reporting EndMT tend to focus on a customized and restricted subset of markers, making it challenging to validate data and signatures across studies, thus underscoring the issue of a consensual molecular definition.

**Figure 2:**
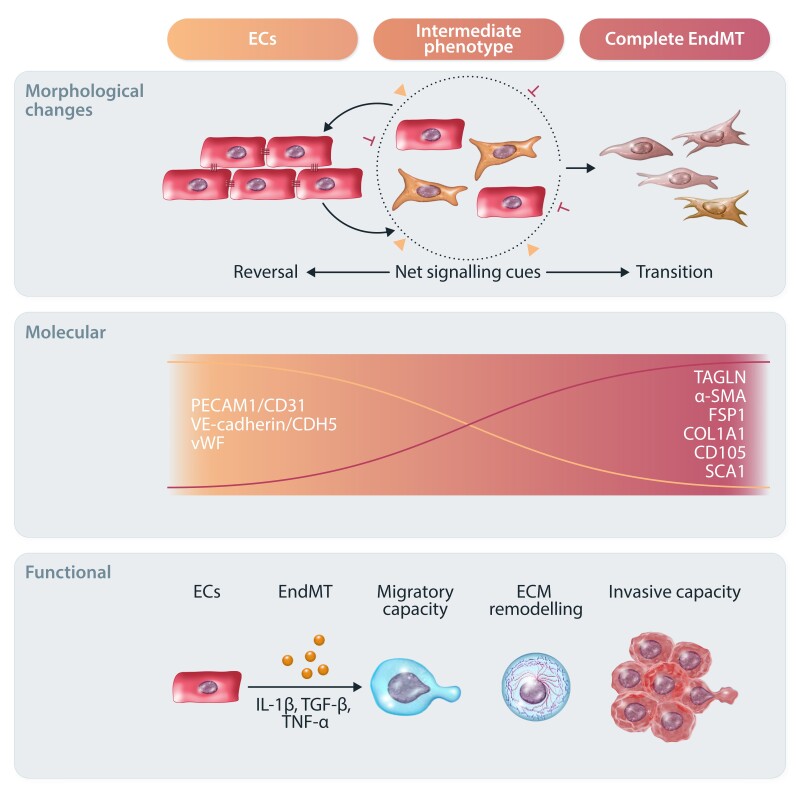
Overview of the EndMT process. Graphical summary of the complex morphological, molecular, and functional changes characterizing EndMT. In response to different stimuli (such as IL-1β, TGF-β, and TNF-α), ECs are activated and undergo EndMT to differentiate towards mesenchymal-like cells. Morphologically, ECs gradually lose their cobblestone structure and cell–cell junctions to acquire an elongated phenotype. This is accompanied by reduced EC-specific marker expression (e.g. CD31, CDH5, and vWF) and increased mesenchymal markers (e.g. TAGLN, α-SMA, FSP-1, COL1A1, CD105, and SCA1). ECs may undergo intermediate or complete EndMT based on net signalling cues. Intermediate EndMT gives rise to intermediary cells that coexpress endothelial and mesenchymal markers. Ultimately, EndMT-derived mesenchymal and mesenchymal-like cells lose most of their endothelial functions and show increased migratory and invasive capacities.

EC morphology changes such as cytoskeletal remodelling, loss of cell–cell junctions, and loss of cellular polarity are broadly reported in the literature.^53^ Evidence shows that during early stages of EndMT there is a decrease in intercellular adhesion forces accompanied by increases in cellular stiffness.^67^ These changes cause ECs to lose their characteristic cobblestone-like morphology to acquire an elongated shape.^68^ Following cytoskeletal re-arrangements, ECs gain proliferative, migratory, and invasive properties.^15,69,70^  *Figure 2* summarizes the major changes associated with EndMT.

### 3.2 EndMT dynamics

While initial descriptions of EndMT in the context of embryonic development supported the idea that the conversion of ECs to mesenchymal cells was permanent, recent evidence suggests that this process of cellular trans-differentiation is flexible. Indeed, it is now widely accepted that cells undergoing EndMT evolve through various intermediate stages, indicating that this process exists in a continuum and that ECs can remain in an intermediate stage and only undergo partial EndMT.^33,71,72^

In partial/intermediate EndMT, both endothelial and mesenchymal features may be present, whereas in a more complete transition, cells are suggested to reach a mesenchymal state. In PAH, lineage tracing enabled the identification of both partial and complete EndMT states with distinct marker profiles.^73^ In this study, the partial EndMT cells expressed endothelial progenitor cell markers such as prominin-1 (PROM1/CD133) and CD34, while fully transitioned cells coexpressed mesenchymal stem cell markers such as the stem cell antigen 1 (SCA1) and endoglin (ENG/CD105).^73^ Of note, ENG is expressed in vascular ECs but it further increases in mesenchymal-like cells. As such, it is used as one of a number of mesenchymal markers when defining endothelial or EndMT identity, and it is evaluated in terms of relative expression changes as opposed to its presence or absence. In the same PAH study, cells that had undergone complete EndMT also showed enhanced proliferative and migratory capacity; however, there was a reliance on only a limited set of markers to identify such properties. A recent lineage tracing study in a myocardial infarction (MI) model reported that EndMT genes were enriched in regions of EC clonal expansion.^56^ The authors described this as partial EndMT because clonally expanded ECs did not lose expression of endothelial genes. In other studies, single-cell transcriptomic and epigenomic data show a spectrum of intermediate phenotypes.^74,75^ Cell subpopulations that coexpress endothelial and mesenchymal markers have been identified also in cardiac,^76^ pulmonary,^77^ and dermal^78^ fibrosis, as well as in embryonic and adult valve ECs.^71,79,80^ Importantly, it is not understood if the path from partial to complete EndMT is linear or if a group of cells undergoes partial EndMT while others have the capacity to undergo a complete transition. It is important here to also account for EC origins and their significant heterogeneity, which could predispose a certain group of ECs to be more or less likely to undergo EndMT. It is believed, however, that partial EndMT is not a distinct process but rather an incomplete activation and differentiation towards EndMT, where certain signals (or lack thereof) prohibit ECs from fully transitioning towards a mesenchymal phenotype.^81^

While in contexts such as those of embryonic development, EndMT appears to be complete likely due to high cell plasticity that makes ECs more responsive to the prevailing molecular cues. Alternatively, complete EndMT may result from chronic activation of the EndMT signalling network which drives cells through their intermediate states and towards a robust transition to mesenchymal cells. An example of such plasticity are EndMT-derived mesenchymal cells of the endocardial cushions in the forming embryonic heart.^50,58,82^ In angiogenesis, however, it is proposed that EndMT is partial and that there are regulatory cues in place that inhibit progression of the EndMT programme, which supress a complete transition towards a mesenchymal phenotype.^83,84^ Whether this transient state leads to a complete acquisition of mesenchymal cell fates or is resolved seems to be dictated by specific intracellular milieus and pathological stressors. Furthermore, a recent study from Tombor and colleagues^57^ reported an acute transient mesenchymal activation of ECs post-MI, where ECs adopt a mesenchymal signature within 7 days of MI and return to baseline endothelial identity by 14 days of MI, as opposed to fully committing to a mesenchymal cell fate. This transiency, defined as endothelial-to-mesenchymal activation (EndMA), may facilitate regeneration and does not appear to play a causal role in later disease stages. Notably, additional *in vitro* work showed that the removal of conditioning stimuli resulted in reversal from a mesenchymal-like state in cultured ECs. Consistently, independent research showed that decreased expression of receptors involved in transforming growth factor-β (TGF-β)-mediated responses (e.g. ALK2 and ALK5), as well as EndMT-associated TFs such as SNAI1, results in a comparable repression of EndMT in EC cultures.^85^ These observations open new possibilities for the investigation of EndMT reversibility in relevant pathologies. Further studies are warranted to determine the precise mechanisms at play, especially in situations where EndMT acts chronically such as in atherosclerosis.

### 3.3 Integrated signalling pathways and contribution to EndMT

The EndMT programme is orchestrated by a variety of biochemical, biomechanical, and environmental signals and then executed by various signalling cascades together with TFs. In disease, it is advanced by acute/chronic environmental changes, including but not limited to inflammation, hypoxia, and alteration in biomechanical forces as reviewed elsewhere.^85,86^ In particular, inflammation is an intrinsic component of EndMT wherein the inflammatory response is mediated by two key cytokines: interleukin-1 beta (IL-1β) and tumour necrosis factor alpha (TNF-α).

**Figure 3:**
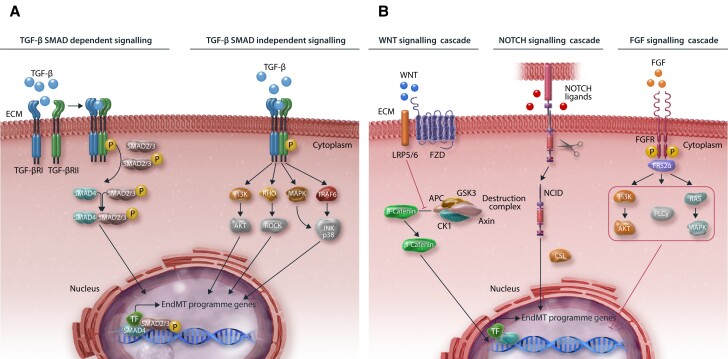
Key signalling pathways involved in EndMT modulation. (*A*) Graphical illustration of TGF-β signalling (*B*) and other common pathways in EndMT. For SMAD-dependent signalling, TGF-β binds to and activates the TGF-β I/II receptor complex. This results in the recruitment of SMAD2/3 proteins, which form a complex together with SMAD4. This is translocated into the nucleus to induce the expression of EndMT-associated genes. SMAD-independent TGF-β signalling pathways include, among others, MAPK, RHO, PI3K, and TRAF6. Other common signalling cascades that regulate EndMT include WNT, NOTCH, and FGF. Binding of WNT to the LRP5/6-FZD receptor complex mediates translocation of β-catenin into the nucleus by promoting de-assembly of the β-catenin destruction complex (APC-GSK3-Axin-CK1). NOTCH signalling activation involves the cleavage, release, and translocation of NICD into the nucleus and subsequent EndMT induction. FGF signalling involves the induction of downstream PI3K, PLCγ, and RAS signalling cascades mediated by FRS2α anchoring to FGFR and subsequent EndMT inhibition.

**Figure 4:**
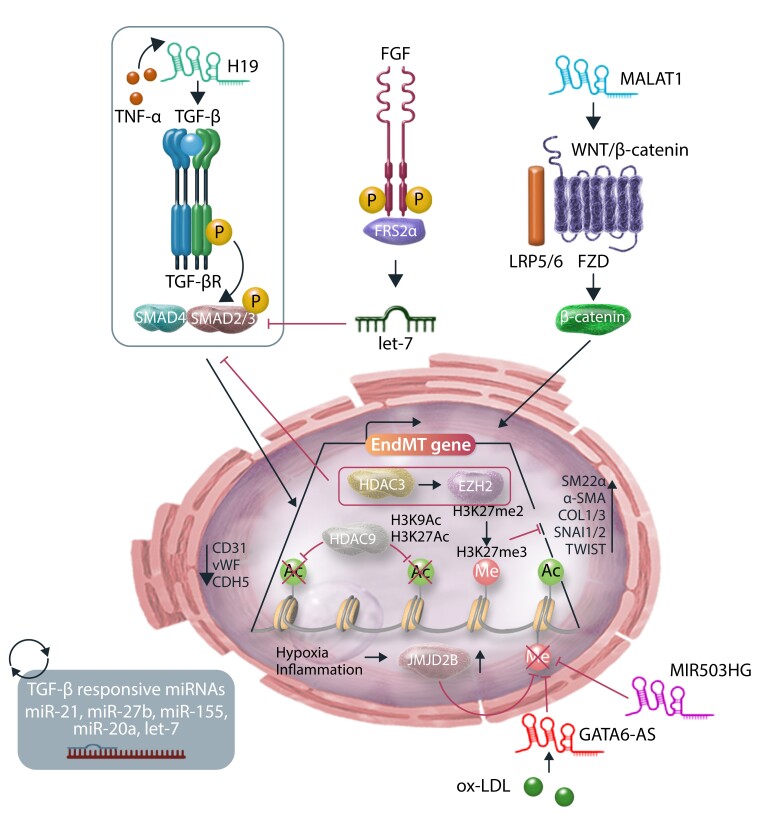
Epigenetic mechanisms and regulation of EndMT. Schematic representation of epigenetic modifications and ncRNA-mediated regulation of EndMT. HDAC3 recruits EZH2 that via H3K27me3 deposition silences TGF-β signalling and blocks EndMT. HDAC9 induces EndMT by repressing H3K9 and H3K27 acetylation. Histone demethylase JMJD2B advances EndMT by demethylating the repressive H3K9me3 at promoters of EndMT controlling genes. LncRNAs H19 and MALAT1 activate the EndMT transcriptional programme by activating the TGF-β and WNT/β-catenin cascades, respectively. LncRNAs GATA6-AS and MIR503HG act by inhibiting EndMT and maintaining EC identity. Many miRNAs have been implicated in regulating EndMT, by either inhibiting or promoting this process, either directly or indirectly. For example, dysregulation of FGF signalling results in reduced let-7 miRNA levels, which in turn increases TGF-β signalling and induces EndMT.

The activated signalling cascades up-regulate a set of TFs promoting acquisition of mesenchymal cell states and EC identity loss. While TGF-β signalling is considered a central aspect of the EndMT programme,^8^ additional pathways also contribute. As EndMT consists of multi-step fate changes, it is finely regulated in differential and sequential manners via inter-connected molecular pathways.^87,88^ In addition, EndMT-associated TFs (e.g. SNAI1 and 2, ZEB1 and 2, and TWIST) can contribute to EndMT being at the intersection of different signalling cascades. However, their action is postulated to be non-redundant and context-dependent as in EMT-associated disorders.^89^ Therefore, distinct ECs may display different TF modulation in terms of preference and timing. Although the precise molecular mechanisms and determinants governing EndMT, at a higher level, remain largely unknown, herein, we discuss the central role of the TGF-β pathway and its independent or synergistic action with other genetic and epigenetic mechanisms implicated in EndMT, with a focus on cardiovascular biology (respectively *Figures 3* and *4*). Causality of these biological processes in EndMT and cardiovascular pathologies is not discussed in detail as current knowledge about their precise contribution is still limited.

The TGF-β superfamily is the major modulator of EndMT processes and comprises an array of responses from direct TGF-β-mediated ones to those depending upon other family members including bone morphogenetic proteins (BMPs) and activins, among others. The canonical TGF-β signalling pathway is known to be a key driver of EndMT.^9,33,40,42,90–92^ While it is essential for correct embryo development, in the adult, EndMT is mostly initiated under pathophysiological cues that lead to aberrant hyperactivation of the TGF-β pathway.^93^ Briefly, TGF-β ligands bind to type I and type II receptors (TGF-βRI and TGF-βRII), which upon heterodimerization and phosphorylation, activate regulatory SMAD2/3 and their intracellular cascades. SMAD2/3 then translocate into the nucleus to form transcriptional complexes that in turn mediate targeted gene activation/repression. Ultimately, the activation of this intracellular cascade regulates several processes including cell differentiation, adhesion, proliferation, migration, and apoptosis that altogether participate in EC activation and transition towards a mesenchymal-like state.^19^ The use of genetically modified mouse models with knockout of TGF-βRs markedly reduces EndMT and kidney fibrosis.^94^ Additionally, in a hyperlipidaemic mouse model of atherosclerosis, TGF-β receptor knockout attenuated EndMT and reduced vessel wall inflammation and permeability with a subsequent arrest of disease progression and regression of the established lesions.^42^ TGF-β also promotes tissue fibrosis by ECM deposition and scavenging, and via the positive modulation of fibrogenic genes such as fibronectin and some collagen species (e.g. COL1A1 and COL3A1).^95^ Finally, MEKK3 is a known mediator involved in EndMT promotion in cavernous cerebral malformations as a result of CNN impairment.^23^ It has also been shown to play an unexpected role in cardiovascular disease by acting downstream of the TGF-β pathway. In more detail, MEKK3 disruption in the endothelium is involved in inward remodelling and promotes EndMT by supporting TGF-β and SMAD2/3 signalling, also correlating with more unstable and vulnerable atherosclerotic plaques.^96^ As an alternative, TGF-β non-canonical pathways act in a SMAD2/3-independent manner, signalling via mediators like the mitogen-activated protein kinase (MAPK) family of serine/threonine-specific protein kinases, phosphatidylinositol 3-kinase (PI3K), p38 MAPK, Jun amino-terminal kinase (JNK), and ubiquitin ligase TNF receptor–associated factor 6 (TRAF6), among others. Transduction of non-canonical signalling cascades occurs in a context-dependent manner to fine-tune specific biological processes.^58^ While there is no compelling evidence to date, it is conceivable that non-canonical and canonical responses may alter SMAD-dependent signalling, thereby contributing to shape the final impact of TGF-β on the system.

BMP-ligand interactions and receptor binding mediate signal transduction via SMAD-dependent and SMAD-independent pathways. This is important, as balance in the BMP/TGF-β axis seems to help maintain cell homeostasis by preventing excessive TGF-β sensitization.^93,97^ BMP signalling has been implicated in osteogenic differentiation and mineralization,^98,99^ which are consistent with EndMT. For instance, the down-regulation of the BMP type II receptor (BMPR2) is a key event in EndMT activation and vascular calcification.^99^

Interestingly, similar to the TGF-β canonical pathway, activins induce a TGF-βRI activation thus triggering SMAD2/3-dependent responses. In PAH, activin A has been shown to promote EndMT by acting as a ligand for BMPR2 and targeting it for degradation.^100^

Lastly, the TGF-β pathway is tightly interconnected to other EndMT signalling cascades that sustain or halt its action, as described below.

In embryo development, canonical wingless-related integration site (WNT) signalling is required for EndMT in the endocardial cushions of the developing heart.^47^ In contrast, in adult life, pathway modulation has also been linked to EndMT in a variety of aberrant tissue remodelling scenarios.^92,101^ In healthy endothelium, the WNT canonical pathway is inactive and expression levels of its primary effector, namely β-catenin, are negatively regulated by the destruction complex (APC-GSK3-Axin-CK1). Upon activation, frizzled (FZD) receptors bind to their coreceptor, low-density lipoprotein (LDL) receptor–related protein 5/6 (LRP5/6), to trigger β-catenin intracellular changes. After translocation to the nucleus, β-catenin promotes LEF/TCF-mediated transcriptional regulation. A post-MI study demonstrated that WNT pathway activity contributes to cardiac fibrosis by expanding αSMA-positive myofibroblasts.^101^ This is in line with increased expression of markers like SNAI2.^102^ Additionally, an independent study showed that inhibition of WNT signalling via the secreted frizzled-related protein 3 (sFRP3) blocks EndMT in mitral valves post-MI. sFRP3 plasma levels were negatively correlated with the size of MI and positively correlated with ejection fraction in sheep with MI.^103^ When promoting EndMT, the WNT and TGF-β signalling cascades can synergize via β-catenin that upon translocation interacts with LEF/TCF and forms SMAD-associated transcriptional complexes to transcriptionally regulate shared target genes. However, WNT3a and WNT7a have an opposite effect on EndMT.^48^

NOTCH signalling contributes to EndMT initiation in developmental and pathological conditions,^83,84^ acting independently or in concert with the TGF-β pathway.^104^ NOTCH signalling is initiated by ligand binding, which induces proteolytic cleavage of the transmembrane receptor and release of the NOTCH intracellular domain (NICD). NICD then translocates into the nucleus and associates with CBF1/Suppressor of Hairless/Lag1 (CSL), thereby recruiting coactivators to regulate transcription. NOTCH can directly activate TWIST1 expression and facilitates the recruitment of SMAD3 to SMAD-binding elements in the promoter of SNAI2 and other mesenchymal genes.^87,105^ Additionally, Jagged1 (JAG1)-induced activation of NOTCH signalling results in the nuclear accumulation of the mesenchymal transcription factor RUNX3 that induces the expression of several mesenchymal genes.^104,106^

Ligands of the fibroblast growth factor (FGF) family bind to FGF receptors (FGFRs), promoting their dimerization and trans-phosphorylation of specific tyrosine residues in cytoplasmic kinase domains, thus triggering their activation.^48^ The additional constitutively docked adaptor protein FGF receptor substrate 2 alpha (FRS2α) generates docking sites for numerous cytoplasmic proteins. As a result, it can trigger an array of mediators and downstream signalling cascades, including phospholipase C gamma (PLCγ), PI3K/AKT, and Ras/MAPK responses.^47,91^ FGF2 (or basic FGF, bFGF) participates in the maintenance of EC functionality by hindering TGF-β-mediated responses.^107,108^ Accordingly, further research has also demonstrated that impaired signalling activity either at the receptor level (FGFR1) or of intracellular transduction (FRS2α) can lead to induction of TGF-β signalling and EndMT as assessed by increased neointima formation in transplant arteriopathy and atherosclerosis models.^90,91^ Mechanistically, active FGFR1 recruits FRS2α, which then induces the expression of microRNA (miRNA, miR) let-7 and suppresses TGF-βRI expression.^90^ Interestingly, FGFR1 expression levels in ECs are negatively affected by pro-inflammatory stimuli, including TNF-α and IL-1β and interferon-γ (IFN-γ), which further impair FGF.^19^ Reportedly, a similar miRNA/mRNA axis has been described for miR-20a.^107^ FGFR1/2 impairment was also shown to increase TGF-β signalling and EndMT in response to hypoxic stimulation both in cultured ECs and in a murine model of pulmonary hypertension.^109^ Finally, FGF has been postulated to play an important role in the maintenance of the vascular endothelial growth factor (VEGF) pathway by supporting VEGF receptor 2 (VEGFR2) expression, which is an important determinant of EC identity through FGF-Ras-MAPK signalling.^48^

### 3.4 Epigenetic orchestration of EndMT

Epigenetic mechanisms can guide gene expression by regulating transcription independently of the genetic code.^22,110^ The term epigenetics refers to those heritable changes that, rather than relying on alteration of the DNA sequence, influence chromatin structure and thereby gene expression. Epigenetic changes are principally based on chemical alterations of DNA (e.g. methylation), post-transcriptional modification of histone proteins (e.g. methylation, acetylation, and phosphorylation), or specific modifications driven by non–protein-coding RNAs (ncRNAs).^110–112^ An increasing number of reports have implicated epigenetics in EndMT, thus adding another layer of regulation to the mechanisms facilitating or inhibiting EndMT biology. As shown in *Figure 4*, here, we aim to provide an outlook of such epigenetic mechanisms; however, this topic has been dissected in depth by recent work.^113–115^

### 3.5 Epigenetic marks

The class I histone deacetylase (HDAC) member termed HDAC3 and the enhancer of zeste homolog 2 (EZH2) play an important role in EC function and in the EndMT programme, both during development and post-natally.^48,116,117^ Briefly, EZH2 is recruited by HDAC3 to promote deposition of tri-methylation marks on the lysine 27 residue of histone 3 (H3K27me3), thereby mediating negative transcriptional regulation of EndMT by repressing TGF-β1 during cardiac development.^116^ Additionally, loss of EZH2 and impaired H3K27me3 deposition at the promoter of TAGLN results in EndMT promotion.^117^ Further exploring the link between HDACs modulation and EndMT, Lecce and colleagues^34^ showed that HDAC9, a class IIa HDAC, positively regulates EndMT both under cytokine-evoked stimulation in cultured ECs and in a murine model of atherosclerosis. HDAC9 exerts its role in EndMT by repressing H3K9 and H3K27 acetylation and preventing the increase in H3K27 methylation. Accordingly, the histone demethylase jumonji domain-containing protein 2B (JMJD2B) is induced by EndMT in response to pro-inflammatory or hypoxic stimuli, advancing EndMT programme activation. While its disruption affects EndMT in an MI model, further studies are needed to define its potential benefits in heart disease.^118^ As another example, the epigenetic reader bromodomain-containing protein 4 (BRD4) has also been described as an EndMT inducer. Mechanistically, it supports EndMT-associated TFs by promoting enhancer occupancy and activation of SMAD-dependent TGF-β responses.^119,120^

### 3.6 Non-coding RNAs

A wealth of research has validated the importance of ncRNAs in different pathologies, including those affecting the vasculature,^110^ the endothelium, and EndMT.^22,114,121^ Based on their size, ncRNAs can be classified as miRNAs and long-ncRNAs (lncRNAs), ranging from ∼22 nucleotides in length to >200 nucleotides, respectively. At present, knowledge on lncRNA contributions to EndMT remains limited. However, among the TGF-β-responsive miRNAs studied in the context of EndMT, many have been implicated including miR-21, miR-27b, miR-155, miR-20a, and let-7.^90,107,114,115,122^

The metastasis-associated lung adenocarcinoma transcript 1 (MALAT1) is a lncRNA that activates EndMT via the canonical WNT pathway and regulates the miR-145/TGFβR2/SMAD3 axis. Another lncRNA of interest is H19, as its role has been previously reported in other cardiovascular conditions, including endothelial ageing, mineralization of aortic valves, ischaemia/reperfusion-evoked apoptosis, and cardiac hypertrophy.^123^ Mechanistically, H19 is activated in ECs by TNF-α to promote TGF-β signalling transduction via H19/TET-1/let-7 axis modulation.^124^ An additional study has reported a role for H19 in oxidized LDL-induced EndMT via modulation of the H19/mir-148b-3p/ELF5 axis.^125^ The lncRNA GATA binding protein 6-AS (GATA6-AS) is induced by oxidized LDL and suppresses TGF-β2-induced EndMT *in vitro* by targeting lysyl oxidase-like 2 (LOXL2) and regulating its impact on angiogenesis.^115^ Concordantly, recent work from Monteiro and collaborators^35^ has shown that lncRNA MIR503HG is crucial in maintaining EC identity and function. In more detail, MiR503HG interacts with polypyrimidine tract binding protein 1 (PTBP1) to broadly prevent the execution of the EndMT programme, both *in vitro* and *in vivo*. Interestingly, its mechanism of action is independent from miR-424 and miR-503, which are located in the same genomic locus and previously described in EMT.^126,127^

Collectively, a better understanding of the genetic and epigenetic determinants that govern EndMT at a higher level is essential as it may permit a crystallizing of knowledge around key steps in the initiation, advancement, and execution of this programme. This may promote a shift in the current state-of-the-art, creating the necessary knowledge for the development of novel therapeutic approaches.

## 4. EC plasticity potential in EndMT: fate of EndMT-derived cells

The multi-step cell fate changes observed in EndMT can be described as a continuum rather than a binary event.^87,88^ As such, this transition can eventually lead to mesenchymal-like cells where the EC transcriptional signature is no longer present. However, intermediate stages are characterized by coexpression of both endothelial and mesenchymal signatures and can potentially shift between lineages.^81,83,116^ Conceivably, transitioning ECs can re-establish a homeostatic balance, or mesenchymal-like cells can further differentiate into other mature cell types.^23,108^ Considering process dynamics and EC heterogeneity within the backdrop of distinct EndMT stages, it is tempting to speculate that partial or complete activation of the EndMT programme can prime or induce *ex novo* formation of specialized EC subpopulations having a central role in human disease. Additional studies are warranted to better characterize the plasticity of EndMT-responsive EC subsets and define whether a common ‘transitioning signature’ is present and then modulated by local cues or if cells are already primed to follow lineage-specific changes. Additional investigation is also needed to define whether partial EndMT is the main manifestation of this process in the adult (as opposed to complete EndMT). Studying specific local cellular environments is crucial, not only because of the impact it may play on EndMT-responsive cells but also to understand how EndMT may reshape the biology of bystander cells and how it fits into an EndMT continuum model.

### 4.1 Identification and characterization of mesenchymal-derived cells of endothelial origin

The balance between competing cell fates is responsible for determining and maintaining cellular identity.^44,81,128^ For ECs to lose their identity and acquire that of another cell type, they must overcome signalling cues that maintain EC fate.^81^ This can be achieved when mesenchymal signals are strong enough to overcome those of the endothelial phenotype. The balance between these two opposing genetic programmes may guide partial vs. complete EndMT, with the latter representing a disruption of such equilibrium.

It is reported that the response of ECs to EndMT stimuli is highly heterogeneous and dependent on multiple factors, such as length of EndMT induction and local cues.^3,43,45^ Furthermore, different patterns of gene expression have been reported when inducing EndMT in differing EC populations, for example, in human microvascular ECs (HMECs) compared with macrovascular human umbilical vein ECs (HUVECs).^129^ A recent study stemming from our collaborative work also demonstrated that treatment of HUVECs and human pulmonary arterial ECs (HPAECs) with TGF-β2 (10 ng/mL) and IL-1β (1 ng/mL) for 7 days induced EndMT while TGF-β2 or IL-1β alone did not.^35^ Deep RNA sequencing of these cells showed that the effect of EndMT induction was stronger in HUVECs compared with HPAECs. As for the contribution of IL-1β to EndMT, a study showed that IL-1β (10 ng/mL) alone is sufficient to induce EndMT in human coronary arterial cells (HCAECs), even after 24 h of stimulation.^99^ However, previous work suggested that IL-1β supports EndMT initiation, without being essential for its progression and maintenance.^130^ Moreover, it was recently reported that stimulating HMECs with TNF-α induces EndMT in a dose-dependent manner (20–100 ng/mL) after 96 h,^54^ whereas a different study showed no effect of TNF-α on human intestinal microvascular cells even after 6 days of EndMT induction.^131^ Additionally, in PAECs, the combination of TGF-β1 with TNF-α and IL-1β was found to be more powerful for EndMT induction than any of these mediators alone.^40^ Overall, this heterogeneity is mainly attributed to different cell lines and stimulating cytokines *in vitro*.

The response of ECs becomes increasingly complicated when EndMT is induced via different stimulating factors, such as hypoxia, disturbed blood flow, and oxidative stress. Hypoxia is a well-known inducer of tissue fibrosis in various pathological processes, and its involvement with EndMT has been previously reported. Exposure of HCAECs to hypoxia (1% O_2_) for 5 days was shown to decrease smooth muscle marker α-SMA while increasing SM22α levels.^61^ When looking at HPAECs however, oxygen levels were shown to have no effects on α-SMA, compared with baseline levels. Furthermore, in both cell types, the endothelial marker CD31 was up-regulated. Other studies have also reported similar findings in terms of changes in EC markers.^132,133^ Of note, the expression of EC markers may not all occur simultaneously and may also be specific to individual transcripts/proteins. Additionally, it is likely that during partial EndMT, there is minimal initial loss of the EC phenotype, which only occurs once the transition is completed.^132^

When looking at the effects of disturbed blood flow in differing contexts, multiple changes arise such as a transition from atheroprotective phenotypes to pro-inflammatory cells, mesenchymal (EndMT-derived) cells, haematopoietic stem cells, endothelial stem/progenitor cells, and unexpected immune cell-like phenotypes.^74^ In a report studying the effects of oxidative stress on EC phenotype, it was shown that HUVECs exposed to hydrogen peroxide (H_2_O_2_) are converted to myofibroblasts via EndMT. This was confirmed based on reduced levels of CD31 and VE-cadherin and the increased protein levels of fibrotic and ECM markers, such as α-SMA, fibronectin, FSP-1, and COL3A1.^134^ The list of the above studies is in no way exhaustive; however, it accurately conveys that multiple factors, such as EC subtype, as well as the dose and nature of the inducing stimulus, result in heterogeneous EC responses. The lack of a standardized protocol for inducing EndMT *in vitro* to date remains a challenge, and a deeper characterization is required in order to understand the properties of these distinct EC populations and determine the fate of the resulting mesenchymal cells.

Overall, this body of evidence suggests that important cellular populations may reside at specific stages of the endothelial-to-mesenchymal continuum, ranging from cells with dual identity (both endothelial and mesenchymal) to those derived from a directed maturation of mesenchymal-like cells or in virtue of EndMT reversal. Hence, additional work in this direction could open a new realm of therapeutic strategies.

## 5. Current technical challenges and limitations in the investigation of EndMT

### 5.1 Available models and experimental caveats

#### 5.1.1 *In vitro* models

It has been demonstrated that cultured ECs undergo EndMT when exposed to TGF-β (β1 or β2) alone or in combination with additional stimuli.^19,20,34,35^ Indeed, to better replicate environmental cues fostering EndMT, TGF-β is used in combination with IL-1β (inflammation) or H_2_O_2_.^11,20,130^ Additional cytokine combinations include IFN-γ and TNF-α together with IL-1β.^19,57^ As discussed in the previous section, different stimuli combinations may be selected for EndMT induction in different cellular systems, taking also in consideration their susceptibility to specific ligands (e.g. TGF-β1 vs. TGF-β2).^41,92^ Like most cell culture systems aiming to mimic complex biological processes, the use of EndMT-related conditioning stimuli only partially recapitulates the changes happening *in vivo.* Therefore, a coordinated effort is needed to develop more comprehensive cellular models that also account for factors such as local changes in mechanical forces (e.g. laminar vs. disturbed shear stress), altered oxygen supply, and so on. Nonetheless, models solely based on altered flow present different molecular dynamics and signalling cascade activation compared with drug-based approaches. Similarly, oxygen deprivation alone activates different cellular responses. Hence, coupling these culture systems in different combinations may provide a more reliable *in vitro* model of EndMT. While such effort may permit developing a model widely used across different labs and which enables data sharing and cross comparison, one must acknowledge that different culture systems may be a better representation to a specific disease, thus serving different purposes. For instance, radiation can induce EndMT in cultured cells; however, such an approach seems to be appropriate only when describing changes occurring in post-radiation tumour or, perhaps, in relation to deep space flight.^48,135^

#### 5.1.2 *In vivo* models

Many studies have identified EndMT in murine and human diseased tissues by immunostaining approaches assessing coexpression of endothelial and mesenchymal markers. While this may be considered as evidence of EndMT, it cannot provide any information of its precise contribution to disease. Murine models represent a valuable tool to study how complex processes impact natural disease history *in vivo*, providing insights on key events in human disease. With respect to EndMT, a variety of models have been used to study this process in cardiovascular disease.^11^ The detailed description of such findings is beyond the scope of this review; however, we would like to comment on several interesting points.

While EndMT has been reported in numerous publications, the benefit of these findings for the research community is restricted by the use of customized parameters to identify and validate EndMT occurrence in the respective studies. Additionally, caveats in experimental design and absence of lineage-tracing strategies can lead to misrepresentation of EndMT extent and contribution to disease. Finally, EndMT is a highly dynamic process, displaying different kinetics in a disease-specific manner; it is therefore crucial to inspect how the process evolves in different disease stages to determine its contribution to disease evolution and aggravation/resolution.

The use of DNA site recombination systems has permitted exponential advancement in several research areas.^136^ In particular, the use of Cre-lox-mediated genetic lineage tracing strategies enables the insertion of a Cre-recombinase encoding gene in a locus of interest, namely under the control of a cell lineage–specific promoter. To study the EC lineage, promoters such as Cdh5, Tie2, and Pdgf are commonly used. System limitations notwithstanding, the use of inducible Cre-loxP and insertion of reporter genes allows to target and label cells of endothelial lineage in a more precise fashion.^137^ Indeed, the fusion of the oestrogen receptor (ER) with Cre generates an inducible system in which recombination can be spatially restricted to a specific cellular lineage and also controlled in time. On the other hand, the insertion of fluorescent reporter genes (e.g. Rosa26-tdTomato, -GFP, and -YFP) under the Cre promoter enables cell visualization. Therefore, upon system induction, the STOP sequence is excised at the flanking loxP sites by the Cre recombinase, leading to fluorescent reporter expression.^136^ Such labelling is permanent and marks cells of endothelial lineage, regardless of changes in their cellular fate upon pathological challenge. In addition, dual recombinase models can be obtained, for instance, by pairing Cre- with Dre- or Flp-recombinases with a variety of different possible readouts (e.g. combined or exclusive signal). For example, a study by the Zhou lab employed Cdh5-Cre in combination with αSma-Dre to study EndMT and transient αSma activation in ECs during cardiac development.^138^ The use of fate mapping strategies and the study of transition stages in time may represent a valuable tool to better understand the EC transcriptional landscape and the spectrum of changes in EC biology along the entire course of differentiation. This effort may lead to the identification of novel markers associated with early, intermediate, and late stages of EndMT, as well as new cell states arising from EndMT. Considering the benefit for *in vivo* models, deciphering the molecular underpinnings of EndMT would contribute to new conditional murine models and help develop additional lineage tracing strategies by exploiting EndMT-specific markers (endothelial and/or mesenchymal ones) alone or in combination.

### 5.2 EndMT in the multi-omics era

Recent advances in high-throughput RNA sequencing of single cells (scRNA-seq) have enabled the transcriptomic analysis of large number of cells with single-cell resolution, providing insights into the transcriptional signature of individual cells.^7^ Specifically, scRNA-seq of ECs undergoing EndMT has proven to be extremely useful in profiling endothelial and mesenchymal gene expression patterns under different *in vitro* and *in vivo* settings. While heterogeneous EC populations have been observed even under control/homeostatic conditions, such changes are enhanced in transitioning ECs in pathological contexts.^57,74,139,140^ In an *in vivo* mouse study of partial carotid artery ligation, scRNA-seq analysis shows that even under stable blood flow conditions ECs are highly heterogeneous and plastic, resulting in different states of transitioning cells that differentiate to more mesenchymal, immune-like, or haematopoietic cells.^74^ This approach has also been recently adopted to describe EC phenotypic changes and induction of pro-inflammatory EndMT in coronary arteries exposed to oscillatory shear stress.^140^ However, as a whole, the currently available scRNA-seq data sets come with many limitations. The main concerns are the limited number of studies investigating EndMT at the single cell level in general and more specifically for *in vivo* settings. Another limitation is that of a low statistical power, whether it is a low sample number or a low number of single cells. While this can be somewhat explained with EndMT being one of the many processes assessed rather than the main experimental question, it does not allow for confident selection of data sets for further analysis or even for selecting a cluster of interest as a possible EndMT population. Additional study design caveats are linked to limited available datapoints and the lack of a molecular consensus, thus affecting high-throughput data analysis and interpretation. Moreover, the majority of the data sets where EndMT population(s) have been identified were not designed with the aim of investigating and characterizing EndMT but rather focus on confirming its presence and association in a specific disease setting.

Yet, another challenge is the fact that most studies isolate ECs based on cell labelling (e.g. CD31+ cells) and subsequent sorting. While in healthy ECs this potentially yields a pure EC population, when EC homeostasis becomes perturbed, the reliance on specific markers such as CD31 to identify all ECs and EndMT-derived cells may be problematic. As already mentioned, CD31 as well as other markers defining the EC lineage progressively diminish as EndMT advances, making the selection of CD31+ populations challenging. As pointed out by us and others, adoption of genetic lineage tracing may facilitate mapping EC fate changes.^11^ This would also provide a tool to identify contamination with other cell types (e.g. fibroblasts and VSMCs) that, in the absence of genetic tracing tools, deeply complicate the identification and downstream analyses of EndMT populations. Moreover, the lack of robust human and translational proof of concept studies remains a major challenge in the field. A recent meta-analysis of VSMCs undertaken by integrating scRNA-seq data from different mouse models of atherosclerosis with data from human carotid lesion endarterectomy samples identified a close relationship between human and mouse *in vivo* data.^141^ Similar approaches may be adopted for ECs and EndMT studies to enable not only mouse-to-human translatability but also to provide a more comprehensive overview of EndMT and its contribution to disease. Collectively, current strategies provide a powerful resource for those investigating EndMT-associated diseases. However, the exact extent or proportion of identified EndMT-derived cells merits further attention.

Several emerging techniques offer promise in the study of EndMT. For example, multi-omics approaches hold promise for both obtaining new insights and for validating prior findings in EndMT biology. Chromium single-cell assay for transposase accessible chromatin (ATAC)-sequencing, for example, could provide insights into specific cell types/states and their associated gene regulatory mechanisms. Currently in the literature, there is a very limited number of studies performing scATAC-seq in the context of EndMT.^74^ Another important consideration is high-throughput proteomic analyses. Quantitative mass spectrometry may be used to study the proteome of EndMT cells compared with homeostatic ECs. More recently, steps towards high-throughput proteomic analyses and EndMT have been made in both cancer biology and in the cardiovascular field.^142,143^ However, at the present time such high-throughput proteomics and other ‘omics’ studies remain sparse in the literature.

## 6. EndMT as a novel therapeutic target

The remarkable plasticity of ECs and the process of EndMT affords novel but challenging therapeutic opportunities.^59,85^ Molecular therapies targeting altered EndMT-related changes may permit the restoration of desired gene expression patterns and subsequent effects on signalling cascades. Several options have been explored in a preclinical setting, ranging from gene therapy to RNA-based therapeutics. On the other hand, cell therapies could benefit from the knowledge of how to block, guide, or redirect the maturation of EndMT-derived mesenchymal cells. Moreover, considering that EC plasticity is further enhanced in a tissue or disease specific manner, the possibility of modulating specific EC-(sub)populations is also intriguing.^7^ However, while targeted drug-delivery holds substantial promise (e.g. nanoparticles and extracellular vesicles), it requires additional optimization and validation in ECs.

Finally, it is crucial that scientists investigate novel molecular mechanisms and unmet needs in EndMT rather than merely exhausting well mined signalling pathways. Integration of high-throughput technologies, lineage tracing strategies, and generation of new *in vivo* models of disease may provide deeper insight of the higher-level mechanisms and determinants orchestrating EndMT. Herein, we discuss in detail some of these therapeutic opportunities.

**Figure 5:**
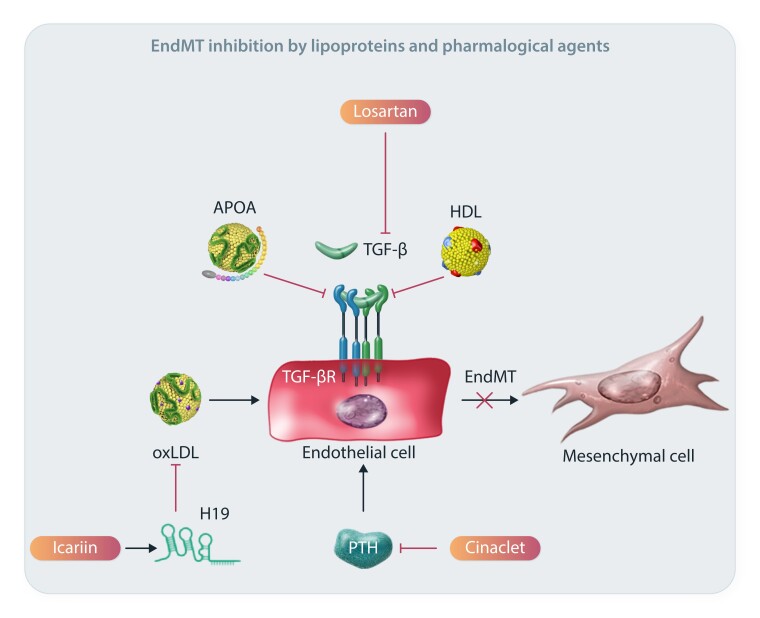
Possible therapeutic avenues of EndMT modulation. Mechanistic details of representative EndMT inhibitors and their associated pathways.

Intensive research has been focused on identifying compounds and drugs that could be used in the clinic as possible EndMT inhibitors. The reported effects are diverse and affect different signalling axes of the EndMT process. For example high-density lipoprotein (HDL) is known to be protective of the endothelium; however, its effects on EndMT are not well studied. The first evidence on the inhibiting effects of HDL on EndMT was provided on HCAECs, where HDL reduced TGF-β1-induced EndMT.^144^ Similarly, apolipoprotein A1 (APOA1) was also shown to alleviate TGF-β1-mediated EndMT in HCAECs, causing an increase in EC markers and a decrease in the EndMT-associated TFs SNAI1 and SNAI2.^145^ A promising compound in atherosclerosis treatment is icariin, a prenylated flavonoid compound that has been shown to inhibit oxLDL-induced EndMT via the H19/mir-148b-3p/ELF5 axis in HUVECs.^125^ LncRNA H19 is modulated upon icariin treatment, leading to attenuation of EndMT and subsequent atheroprotective effects. In a series of studies, it was demonstrated that the use of cinacalcet during renal fibrosis treatment inhibited EndMT, and it was suggested that some of the observed beneficial effects of the normalization of parathyroid hormone levels, such as the reduced aortic wall calcification in uraemic rats, was due to EndMT inhibition.^146^ A graphical representation is provided in *Figure 5*.

Lastly, an obvious target for EndMT would be TGF-β signalling pathways, given their crucial role in inducing and maintaining EndMT. Indeed, several research groups have attempted to inhibit or attenuate TGF-β signalling *in vivo*, with promising initial results.^34,42,96^ Still, in the clinic, this has been met with great challenges and side effects. Not only does TGF-β have pleiotropic effects, but its activation also has different effects in distinct cell types. While its activation promotes EndMT and a proinflammatory status in ECs, it has a positive impact on the biology of other vascular cells such as VSMCs.^42,147^ Because of such limitations, the systemic inhibition of this pathway is not likely to be practical. Indeed, the fact that EndMT is a local process and that systemically administered therapies will affect many cell and tissue types that are unrelated to the endothelium will require careful consideration and reconciliation in the design of possible therapeutic approaches to inhibit EndMT. Nevertheless, losartan, which is an angiotensin receptor blocker that can affect TGF-β signalling, has been shown to prevent TGF-β-induced EndMT by blocking downstream ERK phosphorylation in mitral valve ECs. *In vivo*, losartan-mediated TGF-β inhibition reduced the pro-fibrotic changes that occur in mitral valves post-MI in sheep.^148^

Hence, extensive research is currently focused on the inhibition of downstream EndMT pathways, including but not limited to distinct TGF-β-associated molecules that may lead to the development of new EC-specific treatment options.^55^ Indeed, as discussed in this report, new knowledge on EndMT intermediate changes and EC differentiative potential represent promising paths ahead.

## 7. Future perspectives and final remarks

EndMT is the result of multi-step fate changes rather than a binary event. The precise molecular mechanisms and downstream determinants governing this transition remain to be rigorously investigated, especially when considering EC heterogeneity within the gradation of distinct endothelial-to-mesenchymal transition phases. Dissecting the axis of endothelial and mesenchymal fate programmes during EndMT at different disease stages is of utmost importance. While offering a more precise understanding of EndMT evolution and promoting consensual molecular signatures, it will also provide the mechanistic insight needed to develop novel treatment strategies. Taken together, the aspects discussed here underscore the importance of understanding the molecular and functional attributes of EndMT and their exact associations. The enactment of such a research programme may benefit from collaborative work and the establishment of dedicated consortia.

## Data Availability

No new data were generated or analysed in support of this research.
